# Supplementary material for: LIGHT-SABRE Hyperpolarizes 1-13C-Pyruvate Continuously without Magnetic Field Cycling
Source: J Phys Chem C Nanomater Interfaces. 2023 Apr 4;127(14):6744–53. doi: 10.1021/acs.jpcc.3c01128 (PMC10108362; doi:10.1021/acs.jpcc.3c01128)
Supplement: Supplementary file 1 — jp3c01128_si_001.pdf [file jp3c01128_si_001.pdf]

LIGHT-SABRE Hyperpolarizes 1-^{13}C-Pyruvate Continuously, Without Magnetic Field Cycling

Andrey N. Pravdivtsev[a], Kai Buckenmaier[b], Nicolas Kempf[b], Gabriele Stevanato[f], Klaus Scheffler[c], Joern Engelmann[b], Markus Plaumann[c], Rainer Koerber[d], Jan-Bernd Hövener[a], and Thomas Theis[b],[e],[f]**

[a] Section Biomedical Imaging, Molecular Imaging North Competence Center (MOIN CC), Department of Radiology and Neuroradiology, University Medical Center Kiel, Kiel University, Am Botanischen Garten 14, 24118, Kiel, Germany, E-mail: andrey.pravdivtsev@rad.uni-kiel.de, jan.hoevener@rad.uni-kiel.de
[b] High-Field Magnetic Resonance Center, Max Planck Institute for Biological Cybernetics, Max-Planck-Ring 11, 72076, Tübingen, Germany E-mail: kai.buckenmaier@tuebingen.mpg.de
[c] Department of Chemical Sciences, University of Padova, Via Marzolo 1, 35131 Padova, Italy
[d] NMR Signal Enhancement Group, Max Planck Institute for Multidisciplinary Sciences, Am Fassberg 11, 37077 Göttingen, Germany.
[e] Department for Biomedical Magnetic Resonance, University of Tübingen, Tübingen, Germany
[f] Otto-von-Guericke University, Medical Faculty, Institute of Biometry and Medical Informatics, Leipziger Str. 44, 39120 Magdeburg, Germany
[g] Physikalisch-Technische Bundesanstalt, Department 'Biosignals', Abbestraße 2-12, 10587 Berlin, Germany
[h] Departments of Chemistry and Physics, North Carolina State University, 27695, Raleigh, NC, USA E-mail: ttheis@ncsu.edu
[i] Joint UNC-NC State Department of Biomedical Engineering, North Carolina State University, 27606, Raleigh NC, USA

Contents

1. Theoretical background for LIGHT-SABRE S-2
1.1. LIGHT-SABRE in the resonance case ($v_{\mathrm{RF}} = v^{C}$) S-2
1.2. LIGHT-SABRE in the off-resonance case ($v_{\mathrm{RF}} \neq v^{C}$) S-4
1.3. Average polarization build-up S-5
2. Evaluation of polarization level S-6
3. Sequence parameters S-8
4. Effect of the number of spins on ^{13}C polarization S-9
5. Stability measurements S-10
6. SABRE-SHEATH field dependence and optimal field condition S-11
7. ^{13}C-SABRE spectra at the ultra-low field and different exchange regimes S-12
8. References S-13

1. Theoretical background for LIGHT-SABRE

LIGHT-SABRE Hamiltonian for HH'C spin system corresponds to AA'X system using Pouple notations. We use here HH'C symbols instead to avoid confusion with X, Y, Z symbols used for the axes. The Hamiltonian of this system in the rotating frame of reference is as follows:

$$\hat{\mathrm{H}} = -v^{\mathrm{H}}\left(\hat{I}_{\mathrm{Z}}^{\mathrm{H}} + \hat{I}_{\mathrm{Z}}^{\mathrm{H}'}\right) + (v_{\mathrm{RF}} - v^{\mathrm{C}})I_{\mathrm{Z}}^{\mathrm{C}} + v_1^{\mathrm{C}} I_X^{\mathrm{C}} + J^{\mathrm{H}}\left(\hat{\mathbf{I}}^{\mathrm{H}} \cdot \hat{\mathbf{I}}^{\mathrm{H}'}\right) + \\ +2\Sigma\left(\hat{I}_{\mathrm{Z}}^{\mathrm{H}} + \hat{I}_{\mathrm{Z}}^{\mathrm{H}'}\right)\hat{I}_{\mathrm{Z}}^{\mathrm{C}} + 2\Delta\left(\hat{I}_{\mathrm{Z}}^{\mathrm{H}} - \hat{I}_{\mathrm{Z}}^{\mathrm{H}'}\right)\hat{I}_{\mathrm{Z}}^{\mathrm{C}} \qquad \text{(eq S1)}$$

where $v = \gamma B_0(1+\delta)/2\pi$ is the Larmor precession frequency of the corresponding nucleus, $\Delta = \frac{J^{\mathrm{CH}} - J^{\mathrm{CH}'}}{4} = \frac{\Delta J^{CH}}{4}$, $\Sigma = \frac{J^{\mathrm{CH}} + J^{\mathrm{CH}'}}{4}$, v_1^{C} is the amplitude of the RF pulse (in units Hz for ^{13}C) applied at v_{RF} frequency. Here we assumed that $|v_{\mathrm{RF}} - v^{\mathrm{H}}| \gg v_1^{\mathrm{H}}$ hence we neglected the effect of RF pulse on ^{1}H energy levels.

1.1. LIGHT-SABRE in the resonance case ($v_{\mathrm{RF}} = v^{\mathrm{C}}$)

If the ^{13}C RF pulse is applied exactly in resonance with ^{13}C, then the Hamiltonian structure is significantly simplified. To evaluate the energy levels, it is convenient to transform the axes into a tilted frame using the following axes' rotation for the ^{13}C spin around the Y axis:

$$\begin{aligned} X &\to Z' \\ Y &\to Y' \\ Z &\to -X \end{aligned} \qquad \text{(eq S2)}$$

Then the Hamiltonian in this selectively tilted rotating frame of reference is:

$$\hat{\mathrm{H}} = -v^{\mathrm{H}}\left(\hat{I}_{\mathrm{Z}}^{\mathrm{H}} + \hat{I}_{\mathrm{Z}}^{\mathrm{H}'}\right) + v_1 I_{\mathrm{Z}'}^{\mathrm{C}} + J^{\mathrm{H}}\left(\hat{\mathbf{I}}^{\mathrm{H}} \cdot \hat{\mathbf{I}}^{\mathrm{H}'}\right) + 2\Sigma\left(\hat{I}_{\mathrm{Z}}^{\mathrm{H}} + \hat{I}_{\mathrm{Z}}^{\mathrm{H}'}\right)\hat{I}_{\mathrm{X}'}^{\mathrm{C}} - 2\Delta\left(\hat{I}_{\mathrm{Z}}^{\mathrm{H}} - \hat{I}_{\mathrm{Z}}^{\mathrm{H}'}\right)\hat{I}_{\mathrm{X}'}^{\mathrm{C}} \text{(eq S3)}$$

The convenient basis for this Hamiltonian will be the S-T_0 basis for two protons and the Zeeman basis in the tilted frame for the ^{13}C. In the beginning, only the singlet state $|S\rangle$ of two protons is populated. Therefore for spin order transfer, only $|S\alpha'\rangle$ and $|S\beta'\rangle$ and all other coupled states are of interest. α' and β' indicate the spin states of ^{13}C that are parallel (+½) and antiparallel (projections -½) to the RF field in a rotating frame of reference. This relevant block in the Hamiltonian (eq S3) is then:

$$\hat{\mathrm{H}}_{S\alpha',T_0\beta',S\beta',T_0\alpha'} = \begin{pmatrix} -\frac{3J^H}{4} + \frac{v_1}{2} & -\Delta & 0 & 0 \\ -\Delta & +\frac{J^H}{4} - \frac{v_1}{2} & 0 & 0 \\ 0 & 0 & -\frac{3J^H}{4} - \frac{v_1}{2} & -\Delta \\ 0 & 0 & -\delta & +\frac{J^H}{4} + \frac{v_1}{2} \end{pmatrix} \begin{vmatrix} S\alpha' \\ T_0\beta' \\ S\beta' \\ T_0\alpha' \end{vmatrix} \qquad \text{(eq S4)}$$

Hamiltonian is perfectly block-diagonal with the following blocks:

$$\mathrm{H}_{S\alpha',T_0\beta'} = \begin{pmatrix} -\frac{3J^H}{4}+\frac{v_1}{2} & -\Delta \\ -\Delta & +\frac{J^H}{4}-\frac{v_1}{2} \end{pmatrix} \Big| \begin{matrix} S\alpha' \\ T_0\beta' \end{matrix} \text{ and } \mathrm{H}_{S\beta',T_0\alpha'} = \begin{pmatrix} -\frac{3J^H}{4}-\frac{v_1}{2} & -\Delta \\ -\Delta & +\frac{J^H}{4}+\frac{v_1}{2} \end{pmatrix} \Big| \begin{matrix} S\beta' \\ T_0\alpha' \end{matrix}$$

(eq S5)

From this, the level anticrossing (LAC) conditions follow:

$$\begin{aligned} S\alpha' - T_0\beta' &: v_1 = J^H \\ S\beta' - T_0\alpha' &: v_1 = -J^H \end{aligned}$$ (eq S6)

When the LAC conditions are met (e.g. $v_1 = J^H$) then the corresponding blocks of the Hamiltonian are as follow:

$$\widehat{\mathrm{H}}_{S\alpha',T_0\beta'}(v_1 = J^H) = \begin{pmatrix} -\frac{J^H}{4} & -\Delta \\ -\Delta & -\frac{J^H}{4} \end{pmatrix} \Big| \begin{matrix} S\alpha' \\ T_0\beta' \end{matrix}$$ (eq S7)

$$\widehat{\mathrm{H}}_{S\beta',T_0\alpha'}(v_1 = J^H) = \begin{pmatrix} -\frac{3J^H}{4}-\frac{v_1}{2} & -\Delta \\ -\Delta & +\frac{J^H}{4}+\frac{v_1}{2} \end{pmatrix} \Big| \begin{matrix} S\beta' \\ T_0\alpha' \end{matrix}$$ (eq S8)

Eigenvalues and vectors for the Hamiltonian block $S\alpha', T_0\beta'$ (eq S7) are:

$$\begin{aligned} E_1 &= -\frac{J^H}{4} - \Delta,\ |1\rangle = \frac{|S\alpha'\rangle + |T_0\beta'\rangle}{\sqrt{2}} \\ E_2 &= -\frac{J^H}{4} + \Delta,\ |2\rangle = \frac{|S\alpha'\rangle - |T_0\beta'\rangle}{\sqrt{2}} \end{aligned}$$ (eq S9)

With the energy difference of $E_1 - E_2 = -2\Delta = -\frac{J^{\mathrm{CH}} - J^{\mathrm{CH}'}}{2}$. Finally, the polarization along the Z' axis and this LAC is the following:

$$P_{Z'} = p_{\alpha'} - p_{\beta'} = p_{S\alpha'} - p_{S\beta'} - p_{T_0\beta'}$$ (eq S10)

Here capital P is polarization and small p is the population of the corresponding state. Only populated states are given here. Because the total sum of populated levels is 1, $p_{S\beta'} = \frac{1}{2}$ and $p_{T_0\beta'} = \frac{1}{2} - p_{S\alpha'}$ this equation simplifies to the following:

$$P_{Z'} = -2p_{T_0\beta'}$$ (eq S11)

Using the Schrödinger equation, it is straightforward to find the population of the level $T_0\beta'$ if the system started in the state $\frac{|S\alpha'\rangle + |S\beta'\rangle}{\sqrt{2}}$. Doing so, the polarization generated along the Z' axis finally can be calculated as

$$P_{Z'}(t_{CW}) = -2\left|\left\langle T_0\beta'\left|e^{-i\widehat{\mathrm{H}}t_{CW}}\right|\frac{S\alpha'}{\sqrt{2}}\right\rangle\right|^2 = -\left|\frac{1}{2}\left(e^{-iE_1t_{CW}} - e^{-iE_2t_{CW}}\right)\right|^2 = -\left|\frac{1}{2}\left(e^{+it_{CW}\Delta} - e^{-it_{CW}\Delta}\right)\right|^2 = -\sin^2(2\pi t_{CW}\Delta) \quad \text{(eq S12)}$$

Where again $\Delta = \frac{J^{\mathrm{CH}} - J^{\mathrm{CH}'}}{4}$. In this case, according to eq S2, $P_{Z'} = P_X$ and the hyperpolarized signal can be measured immediately after the LIGHT-SABRE pulse without additional signal excitation.

In the same vein, the polarization in the off-resonance can be calculated.

1.2. LIGHT-SABRE in the off-resonance case ($v_{\mathrm{RF}} \neq v^{\mathrm{C}}$)

In this case, it is convenient to transform the axes in the tilted frame where Z' will be along the effective magnetic field: $\vec{v} = (v_1, 0, v_{\mathrm{RF}} - v^{\mathrm{C}})$ with $\tan(\theta) = \frac{v_1}{v_{\mathrm{RF}} - v^{\mathrm{C}}}$ and $v = \sqrt{(v_{\mathrm{RF}} - v^{\mathrm{C}})^2 + v_1^2}$. It can be achieved using the following rotation for the ^{13}C spin around Y axis:

$$\begin{gathered} X \rightarrow \cos\theta\, X' + \sin\theta\, Z' \\ Y \rightarrow Y' \\ Z \rightarrow \cos\theta\, Z' - \sin\theta\, X' \end{gathered} \quad \text{(eq S13)}$$

Then the Hamiltonian of this system in the rotating frame of reference is:

$$\widehat{\mathrm{H}} = -v^{\mathrm{H}}\left(\hat{I}_{\mathrm{Z}}^{\mathrm{H}} + \hat{I}_{\mathrm{Z}}^{\mathrm{H}'}\right) + vI_{\mathrm{Z}'}^{\mathrm{C}} + J^{\mathrm{H}}\left(\hat{\mathbf{I}}^{\mathrm{H}} \cdot \hat{\mathbf{I}}^{\mathrm{H}'}\right) + +2\Sigma\left(\hat{I}_{\mathrm{Z}}^{\mathrm{H}} + \hat{I}_{\mathrm{Z}}^{\mathrm{H}'}\right)\left(\cos\theta\, I_{\mathrm{Z}'}^{\mathrm{C}} - \sin\theta\, I_{\mathrm{X}'}^{\mathrm{C}}\right) + 2\Delta\left(\hat{I}_{\mathrm{Z}}^{\mathrm{H}} - \hat{I}_{\mathrm{Z}}^{\mathrm{H}'}\right)\left(\cos\theta\, I_{\mathrm{Z}'}^{\mathrm{C}} - \sin\theta\, I_{\mathrm{X}'}^{\mathrm{C}}\right) \quad \text{(eq S14)}$$

Here, $\left(\hat{I}_{\mathrm{Z}}^{\mathrm{H}} + \hat{I}_{\mathrm{Z}}^{\mathrm{H}'}\right)|S\rangle = 0$ while the element $\hat{I}_{\mathrm{Z}}^{\mathrm{H}} - \hat{I}_{\mathrm{Z}}^{\mathrm{H}'}$ mixes *S* and *T*0 states only. It means that we have to consider the same block in the Hamiltonian as in (eq S4):

$$\widehat{\mathrm{H}}_{S\alpha',T_0\beta',S\beta',T_0\alpha'} = \begin{pmatrix} -\frac{3J^H}{4} + \frac{v}{2} & -\Delta\sin\theta & 0 & \Delta\cos\theta \\ -\Delta\sin\theta & +\frac{J^H}{4} - \frac{v}{2} & -\Delta\cos\theta & 0 \\ 0 & -\Delta\cos\theta & -\frac{3J^H}{4} - \frac{v}{2} & -\Delta\sin\theta \\ \Delta\cos\theta & 0 & -\Delta\sin\theta & +\frac{J^H}{4} + \frac{v}{2} \end{pmatrix} \begin{vmatrix} S\alpha' \\ T_0\beta' \\ S\beta' \\ T_0\alpha' \end{vmatrix} \quad \text{(eq S15)}$$

The equations S13-S15 are equivalent to the one eq S2-4 when $\sin\theta = 1$. Unlike the resonance case, the pairs of spin states can not be grouped, resulting in additional leakage of polarization since now all 4 states are coupled. The LAC conditions are similar to the resonance case (eq S6):

$$\begin{gathered} S\alpha' - T_0\beta' : v = J^H \\ S\beta' - T_0\alpha' : v = -J^H \end{gathered} \quad \text{(eq S16)}$$

Let's consider the first LAC again ($v = J^H$):

$$\widehat{\mathrm{H}}_{S\alpha',T_0\beta',S\beta',T_0\alpha'}(v=J^H)=\begin{pmatrix} -\frac{J^H}{4} & -\Delta\sin\theta & 0 & \Delta\cos\theta \\ -\Delta\sin\theta & -\frac{J^H}{4} & -\Delta\cos\theta & 0 \\ 0 & -\Delta\cos\theta & -\frac{5J^H}{4} & -\Delta\sin\theta \\ \Delta\cos\theta & 0 & -\Delta\sin\theta & +\frac{3J^H}{4} \end{pmatrix}\begin{vmatrix} S\alpha' \\ T_0\beta' \\ S\beta' \\ T_0\alpha' \end{vmatrix} \quad \text{(eq S17)}$$

Two other general levels stay J^H apart from the LAC and can be ignored for simplicity. So the most important part of the Hamiltonian then is:

$$\widehat{\mathrm{H}}_{S\alpha',T_0\beta'}(v_1=J^H)\begin{pmatrix} -\frac{J^H}{4} & -\Delta\sin\theta \\ -\Delta\sin\theta & -\frac{J^H}{4} \end{pmatrix}\begin{vmatrix} S\alpha' \\ T_0\beta' \end{vmatrix} \quad \text{(eq S18)}$$

Hence the polarization along the effective magnetic field (see eq S13) will be given by:

$$P_{Z'}(t_{CW})=-\sin^2(2\pi t_{CW}\Delta\sin\theta) \quad \text{(eq S19)}$$

Using eq S13, we can find polarization values in the rotating frame of reference:

$$\begin{gathered} P_X=P_{Z'}(t_{CW})\sin\theta=\sin^2(2\pi t_{CW}\Delta\sin\theta)\sin\theta \\ P_Z=P_{Z'}(t_{CW})\cos\theta=-\sin^2(2\pi t_{CW}\Delta\sin\theta)\cos\theta \end{gathered} \quad \text{(eq S20)}$$

P_X can be observed immediately after the LIGHT-SABRE pulse, for P_Z one has to apply a 90° RF pulse first.

1.3. Average polarization build-up

Finally, let's estimate the amount of polarization transferred during LIGHT-SABRE. The probability of the complex dissociating follows the exponential distribution.

$$dW=k_d e^{-k_d t}dt \quad \text{(eq S21)}$$

Where k_d is the dissociation rate or $\tau_c=1/k_d$ is a lifetime of the complex. Then the average absolute polarization (eq S12) generated in a single event is given by:

$$|\overline{P_{Z'}}|=|\overline{P_X}|=\int_0^{+\infty}\sin^2(2\pi t\Delta)\,dW=\frac{2a^2}{4a^2+1} \quad \text{(eq S22)}$$

With $a=2\pi\frac{\Delta}{k_d}$. $\overline{P_{Z'}}$ is zero for $a=0$ and monotonously grows to 0.5 for $a\to\infty$.

For the typical values of $\Delta J^{CH}=0.1$ Hz and $k_d=10$ s^{-1}, $a=0.0157\ll 1$ hence

$$|\overline{P_X}|\cong 2a^2 \quad \text{(eq S23)}$$

In the same way, the polarization values for the off-resonance case (eq S20) can be found as:

$$|\overline{P_X}| = \frac{2a^2 \sin^2\theta}{4a^2\sin^2\theta+1}\sin\theta \cong 2a^2\sin^3\theta$$
$$|\overline{P_Z}| = \frac{2a^2 \sin^2\theta}{4a^2\sin^2\theta+1}\cos\theta \cong 2a^2\sin^2\theta\cos\theta \quad \text{(eq S24)}$$

Here $|\overline{P_X}|$ reaches the maximum of $2a^2$ when $sin\,\theta = 1$ or $\theta = 90^o$, i.e. in resonance, while $|\overline{P_Z}|$ reaches maximum of $\frac{4}{3\sqrt{3}}a^2 \cong 0.77a^2$ for $\cos\theta = \frac{1}{\sqrt{3}}$ or $\theta \cong 54.7^o$ (magic angle).

It shows that off-resonance conditions are not favorable for polarization transfer when Δ is much smaller than the exchange rate. It is beneficial for polarization when the chemical shifts of bound and free forms are different and one can accumulate the polarization in net magnetization, which is possible only at the off-resonance LIGHT-SABRE conditions.

2. Evaluation of polarization level

In contrast to Faraday coils, a SQUID-based detector is capable of measuring the MR signal in units of fT. If the geometry of a homogeneous sample is known, the polarization level can be calculated[1,2].

First, we need to calculate the detection field strength[3]:

$$B_{\text{det}} = \frac{1}{A_p}\int_{\text{sample}} \beta_\perp(\boldsymbol{r})\, M_{\text{sample}}\, d\text{V},$$

where A_p is the area of the pickup loop, $\beta_\perp(\boldsymbol{r})$ is the component of the received field, which is perpendicular to the precession field and can be calculated analytically as described in Ref. 4, M_{sample} is the thermal equilibrium magnetization which can be calculated via Curie's law $M_{\text{sample}} = \rho_{\text{sample}}\gamma^2\hbar^2 B_p/4k_B T$, where ρ_{sample} is the proton spin density, γ is the gyromagnetic ratio, $\hbar$ is the Planck constant, k_B is the Boltzmann constant, and T is the temperature of the sample.

Therefore, the SABRE reactor was filled with water and the MR signal was measured after prepolarization at $B_p = 0 - 10$ mT for 16 s, via a simple 90° ^{1}H excitation readout scheme. The measured reference detection field strength $B_{\text{det}}^{\text{ref}}$ can now be used for the calibration of $\beta_\perp(\boldsymbol{r})$.

$$M^{1\text{H,ref}} = \frac{\rho_{1\text{H}}^{\text{water}}\hbar\gamma_{1\text{H}}}{2} P\left({}^1\text{H}, B_p, T\right),$$

$$P\left({}^1\text{H}, B_\text{p}, T\right) = \tanh\left(\frac{\hbar\gamma_{1\text{H}}}{2k_B T}\right),$$

hence

$$\frac{1}{A_p}\int_{\text{sample}} \beta_\perp(\boldsymbol{r})\ d\text{V} = \frac{B_{\text{det}}^{\text{ref}}}{M^{1\text{H,ref}}}.$$

For calculating the polarization of 1-^{13}C pyruvate, we need first to know the maximum possible ^{13}C and ^{1}H magnetization for the given sample, which corresponds to magnetization with 100% polarization of the respective spin:

$$M_{\text{max}}^{13\text{C,sample}} = \frac{\rho_{13\text{C}}^{\text{sample}}\hbar\gamma_{13\text{C}}}{2}$$

$$M_{\text{max}}^{1\text{H,sample}} = \frac{\rho_{1\text{H}}^{\text{sample}}\hbar\gamma_{1\text{H}}}{2},$$

where ρ is the spin density of ^{13}C and ^{1}H, γ is the gyromagnetic ratio. Note that $\rho_{1\text{H}}^{\text{sample}} = 3\cdot\rho_{13\text{C}}^{\text{sample}}$, because there is one ^{13}C and 3 ^{1}H atoms in 1-^{13}C pyruvate. The hyperpolarized measured magnetization can be gained out of:

$$M^{\text{sample}} = \frac{B_{\text{det}}^{\text{sample}}}{\frac{1}{A_p}\int_{\text{sample}}\beta_\perp(\boldsymbol{r})\ d\text{V}} = B_{\text{det}}^{\text{sample}}\frac{M^{1\text{H,ref}}}{B_{\text{det}}^{\text{ref}}},$$

with $B_{\text{det}}^{\text{sample}}$ the measured detected field strength.

Hence final polarization can now be calculated as

$$P = \frac{M^{\text{sample}}}{M_{\text{max}}} = \frac{B_{\text{det}}^{\text{sample}}}{B_{\text{det}}^{\text{ref}}}\frac{M^{1\text{H,ref}}}{M_{\text{max}}^{\text{sample}}}.$$

Here $M_{\text{max}}^{\text{sample}}$ and $B_{\text{det}}^{\text{sample}}$ must be used for the same type of nuclei.

Note that this formula can be also applied to calculate multiplet polarization (longitudinal ^{1}H-^{13}C spin order). Because we measured such spin order with ^{1}H excitation and the resulting spectrum consists of two lines only without any overlapping of lines no corrections to the final formula are necessary. The method to calculate multiplet polarization for higher multiplicity see in Ref. 5.

3. Sequence parameters

Table S1. Sequence parameters of the used measurements. All measurements were performed at B_0 = 121 µT and the data acquisition time t_{acq} was 8 s. During the whole experiment, pH_2 was bubbled constantly through the sample at a rate of 2 L/h. Each stability monitoring data point is a result of averaging two consequent repetitions of the SABRE-SHEATH experiment. All other data was acquired without averaging. The hyperpolarization time t_{hyp} was set to 10 s if not varied for accessing the hyperpolarization build-up time T_{hyp}. Here "-Z" in LIGHT-SABRE-Z indicates that the longitudinal component was measured in this experiment.

Measurement	B_{HYP} [µT]	ν_{CW}^{A} [µT]	$\Delta\nu_{CW}^{frq}$ [Hz]	90° pulse	T_{grad} [ms]	τ [ms]	*TR* [s]
**Stability**	0.36	–	–	$^{13}C/^{1}H$	–	–	22.1
**SABRE-SHEATH, B_{hyp}**	0.35–1.23	–	–	$^{13}C/^{1}H$	–	–	22.1
**SABRE-SHEATH, t_{hyp}**	0.36	–	–	$^{13}C/^{1}H$	–	–	132
**LIGHT-SABRE**	B_0	1.1	1297.5	–	–	–	102
**LIGHT-SABRE-Z**	B_0	1.1	1297.5	$^{13}C/^{1}H$	–	–	102
**LIGHT-SABRE, $\Delta\nu_{CW}^{frq}$**	B_0	0.5, 1.1, 1.7	1260–1340	–	–	–	22.1
**LIGHT-SABRE, ν_{CW}^{A}**	B_0	0–4	1285, 1290, 1297.5, 1305, 1310	–	–	–	22.1
**LIGHT-SABRE-Z, $\Delta\nu_{CW}^{frq}$**	B_0	0.5, 1.1, 1.7	1260–1340	$^{13}C/^{1}H$	80	–	22.2
**LIGHT-SABRE-SEPP**	B_0	0.2, 0.5, 1.1, 1.5, 1.7	1260–1340	$^{13}C/^{1}H$	80	208	22.5

4. Effect of the number of spins on ^{13}C polarization

Using the SABRE exchange model presented in the main text, we simulated the effect of adding methyl protons. The results are shown in **Table S2**. The impact of a number of protons is more substantial in the case of SABRE-SHEATH than in LIGHT-SABRE.

Table S2. ^{13}C polarization of pyruvate in the system with different amounts of methyl protons for SABRE-SHEATH and LIGHT-SABRE simulations. The spin system parameters: IrHH *J* coupling constant was -10.5 Hz, only one IrHH proton was coupled with ^{13}C with the interaction of 0.06 Hz, and the interaction of ^{13}C to methyl protons was 1.5 Hz. High field T_1 relaxation times used for simulation of local fluctuating field relaxation superoperator were 0.9 s for IrHH protons, 5 s for methyl protons, and 25 s for ^{13}C. SABRE-SHEATH was prepared at 0.32 μT magnetic field for 120 s. LIGHT-SABRE was prepared at 120 μT magnetic field during 120 s application of in resonance RF pulse with the amplitude 11.8 Hz for ^{13}C. Simulations reported in the main text were done for IrHH-CH_3 spin system.

	τ_{Ir} = 200 ms		τ_{Ir} = 33 ms	
**System**	SABRE-SHEATH: ^{13}C P (%)	LIGHT-SABRE: ^{13}C P (%)	SABRE-SHEATH: ^{13}C P (%)	LIGHT-SABRE: ^{13}C P (%)
**IrHH-C**	7.55	1.8	2.36	1.13
**IrHH-CH**	5.44	1.73	2.19	1.1
**IrHH-CH_2**	4.92	1.65	2.04	1.077
**IrHH-CH_3**	4.1 (54.3% of max)	1.59 (88.3% of max)	1.9 (80.5% from max)	1.05 (92.9% from max)

5. Stability measurements

Due to the continuous bubbling of pH_2 through the solution, the sample evaporated and degenerated over time. This resulted in a decrease in the MR signal. To compensate for this signal loss, a SABRE-SHEATH spectrum was measured (sequence parameters are in **Table S1**) before and after each measurement, so this measurement could be corrected with a scaling factor *s*:

$$s = \frac{(SI_{\mathrm{before}} + SI_{\mathrm{after}})}{2 \cdot SI_{\mathrm{max}}}$$

A summary of all stability measurements is shown in **Figure S1**. The second run decayed faster due to an open lid of the reservoir, leading to an increased evaporation rate.

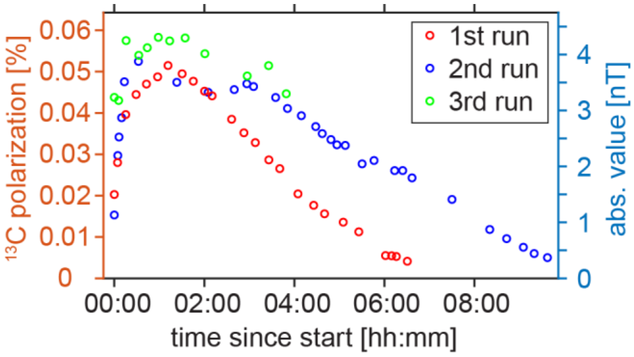

Figure S1. Stability monitoring during experiments. SABRE-SHEATH signal was measured with 10 s of hyperpolarization phase followed by an FID readout. Before and after each measurement point of all presented results the stability was measured. $B_{\mathrm{det}}^{\mathrm{measured}}$ of the ^{13}C signal and the ^{13}C polarization are shown as a function of elapsed time for the 1st, 2nd and 3rd run.

6. SABRE-SHEATH field dependence and optimal field condition

To find the optimal conditions for the SABRE-SHEATH the ^{13}C signal enhancement as a function of hyperpolarization field B_{hyp} was measured (**Figure S2**). Sequence parameters are listed in **Table S1**.

The ^{13}C and ^{1}H spectra were measured simultaneously after 90° ^{1}H, ^{13}C excitation. The ^{13}C signal only raises due to the ^{13}C atoms of 1-^{13}C pyruvate, while the ^{1}H signal is a mixture of ^{1}H signals from 1-^{13}C pyruvate, DMSO, oH_2 and methanol. If and to which degree DMSO or methanol is hyperpolarized was not investigated within this study.

The optimal magnetic field of 0.36 µT corresponds well to the level anticrossing field[6,7]:

$$B_{\mathrm{LAC}} = \left|\frac{2\pi J_{HH}}{\gamma_{1H}-\gamma_{13C}}\right| = 0.33\ \mu\mathrm{T}\ \text{for}\ J_{HH} = -10.5\ \mathrm{Hz}.$$

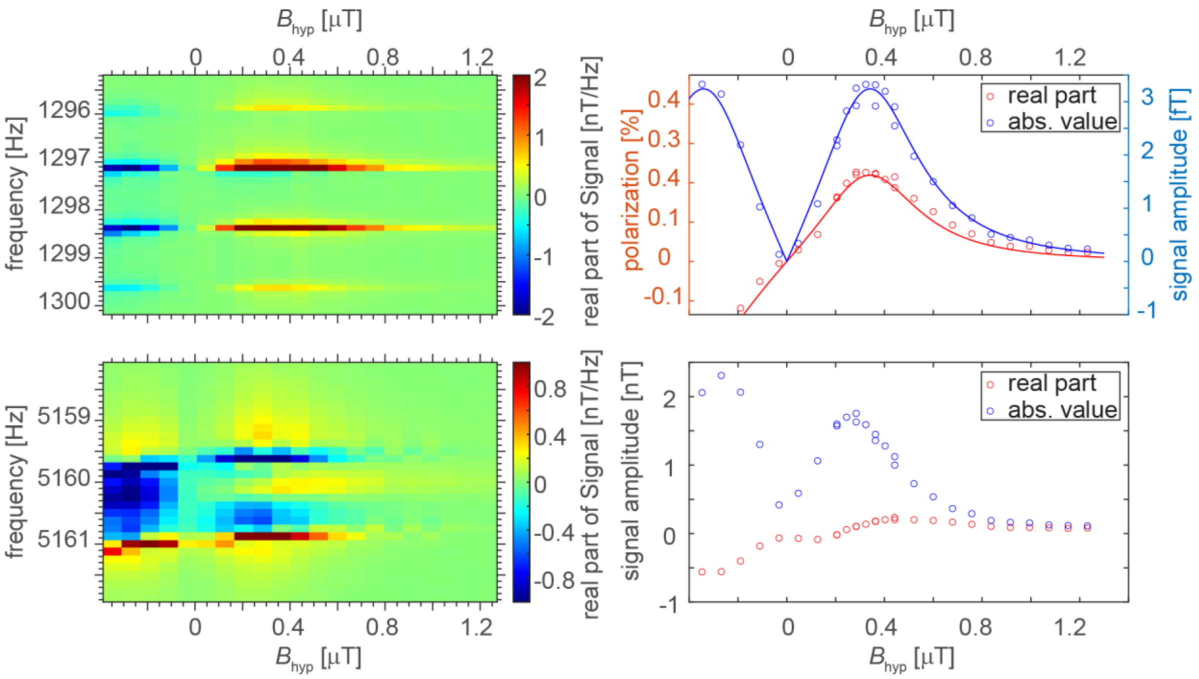

Figure S2. SABRE-SHEATH ^{13}C (top) and ^{1}H (bottom) spectra (left) and integrals (right) as a function of the hyperpolarization field B_{hyp}. Integrals of real part (red) and absolute value (blue).

7. ^{13}C-SABRE spectra at the ultra-low field and different exchange regimes

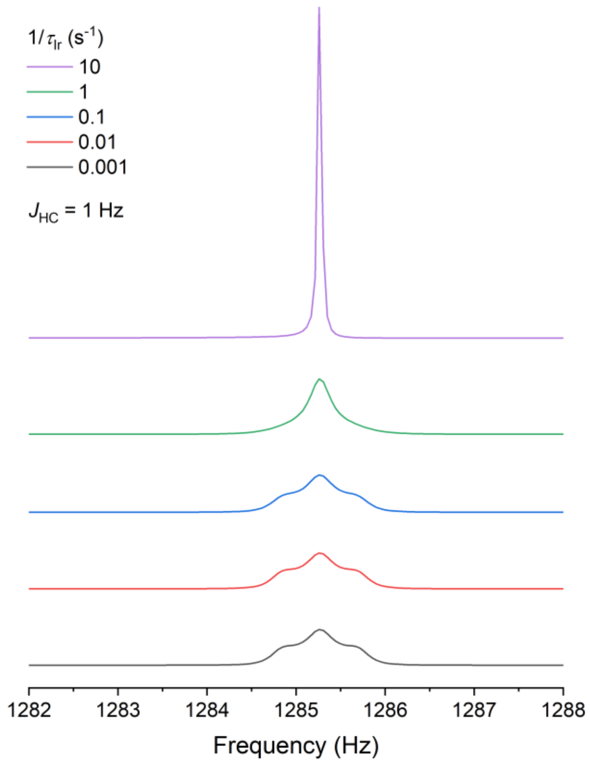

Figure S3. ^{13}C-SABRE spectra of IrHH-C spin system with hyperpolarized ^{13}C for the different lifetimes of the Ir complex τ_{Ir}. The parameters used in the simulation were J_{HC} = 1 Hz (one hydride to ^{13}C, the other coupling is 0), J_{HH} = -10.5 Hz (hydride-hydride). Note that three lines are visible when $1/\tau_{\mathrm{Ir}}$ >> J_{CH}. If, however, it is not the case, $\tau_{\mathrm{Ir}} \leq 1\ s$, then lines collapse into one. One can simulate the case of pyruvate, which has a quartet multiplet. However, it will not change the matter because interactions inside the pyruvate stay the same.

8. References

(1) Buckenmaier, K.; Pedersen, A.; SanGiorgio, P.; Scheffler, K.; Clarke, J.; Inglis, B. Feasibility of Functional MRI at Ultralow Magnetic Field via Changes in Cerebral Blood Volume. *NeuroImage* **2019**, *186*, 185–191. https://doi.org/10.1016/j.neuroimage.2018.10.071.

(2) Buckenmaier, K.; Rudolph, M.; Fehling, P.; Steffen, T.; Back, C.; Bernard, R.; Pohmann, R.; Bernarding, J.; Kleiner, R.; Koelle, D.; et al. Mutual Benefit Achieved by Combining Ultralow-Field Magnetic Resonance and Hyperpolarizing Techniques. *Rev. Sci. Instrum.* **2018**, *89* (12), 125103. https://doi.org/10.1063/1.5043369.

(3) Brown, R. W.; Cheng, Y.-C. N.; Haacke, E. M.; Thompson, M. R.; Venkatesan, R. Magnetic Resonance Imaging: Physical Principles and Sequence Design. In *Magnetic Resonance Imaging*; John Wiley & Sons, Ltd, 2014. https://doi.org/10.1002/9781118633953.ch1.

(4) Simpson, J. C.; Lane, J. E.; Immer, C. D.; Youngquist, R. C.; Steinrock, T. Simple Analytic Expressions for the Magnetic Field of a Circular Current Loop. *NASA Tech. Reports Serv.* **2001**, 1–3.

(5) Pravdivtsev, A. N.; Ellermann, F.; Hövener, J.-B. Selective Excitation Doubles the Transfer of Parahydrogen-Induced Polarization to Heteronuclei. *Phys. Chem. Chem. Phys.* **2021**, *23* (26), 14146–14150. https://doi.org/10.1039/D1CP01891D.

(6) Pravdivtsev, A. N.; Yurkovskaya, A. V.; Vieth, H.-M.; Ivanov, K. L.; Kaptein, R. Level Anti-Crossings Are a Key Factor for Understanding Para-Hydrogen-Induced Hyperpolarization in SABRE Experiments. *ChemPhysChem* **2013**, *14* (14), 3327–3331. https://doi.org/10.1002/cphc.201300595.

(7) Ivanov, K. L.; Pravdivtsev, A. N.; Yurkovskaya, A. V.; Vieth, H.-M.; Kaptein, R. The Role of Level Anti-Crossings in Nuclear Spin Hyperpolarization. *Prog. Nucl. Magn. Reson. Spectrosc.* **2014**, *81* (Supplement C), 1–36. https://doi.org/10.1016/j.pnmrs.2014.06.001.
