# Supplementary figures and images for: LIGHT-SABRE Hyperpolarizes 1-13C-Pyruvate Continuously without Magnetic Field Cycling
Source: J Phys Chem C Nanomater Interfaces. 2023 Apr 4;127(14):6744–53. doi: 10.1021/acs.jpcc.3c01128 (PMC10108362; doi:10.1021/acs.jpcc.3c01128)

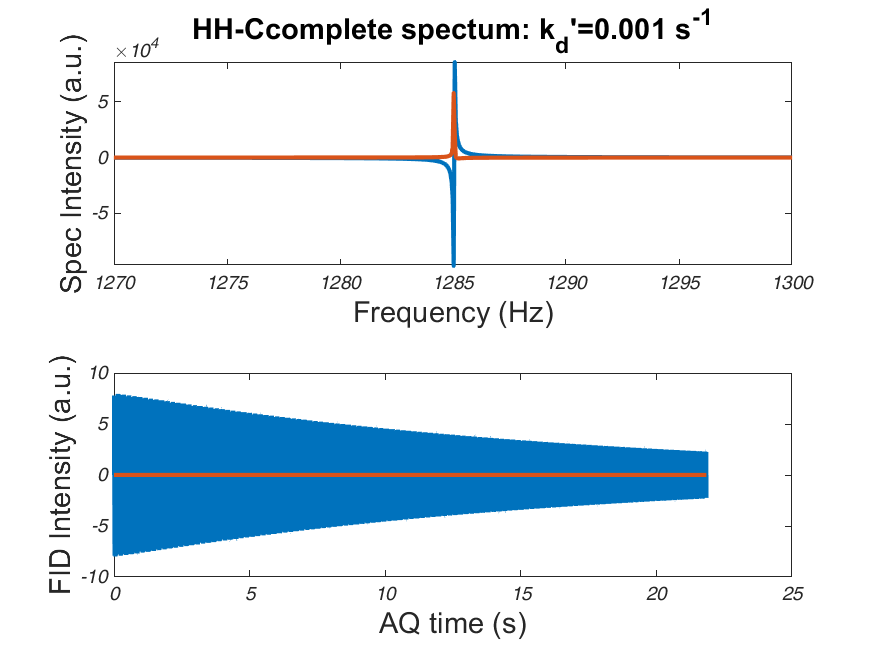

Supplement: Supplementary file 2 — jp3c01128_si_002.zip [file jp3c01128_si_002.zip › LIGHT_SABRE_2023_Paper_Pyruvate_JPCC/out/SLIC_SHEATH_SABRE_HH-C_0.001.png]

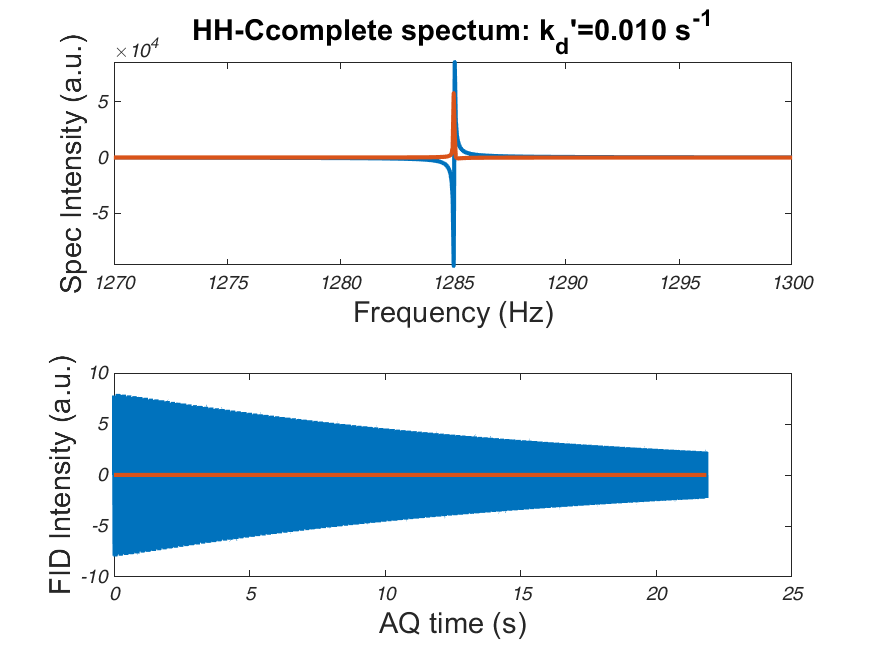

Supplement: Supplementary file 2 — jp3c01128_si_002.zip [file jp3c01128_si_002.zip › LIGHT_SABRE_2023_Paper_Pyruvate_JPCC/out/SLIC_SHEATH_SABRE_HH-C_0.01.png]

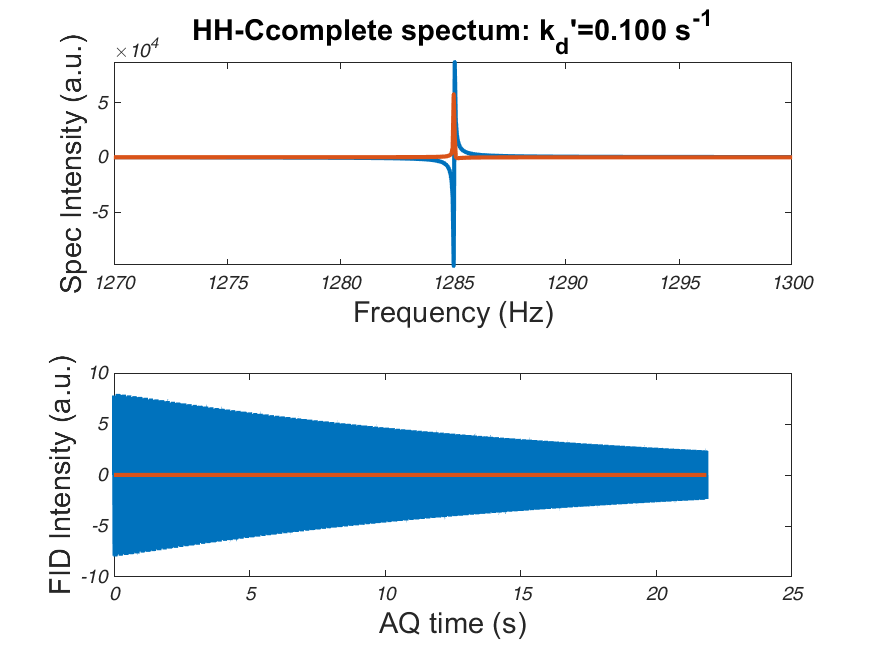

Supplement: Supplementary file 2 — jp3c01128_si_002.zip [file jp3c01128_si_002.zip › LIGHT_SABRE_2023_Paper_Pyruvate_JPCC/out/SLIC_SHEATH_SABRE_HH-C_0.1.png]

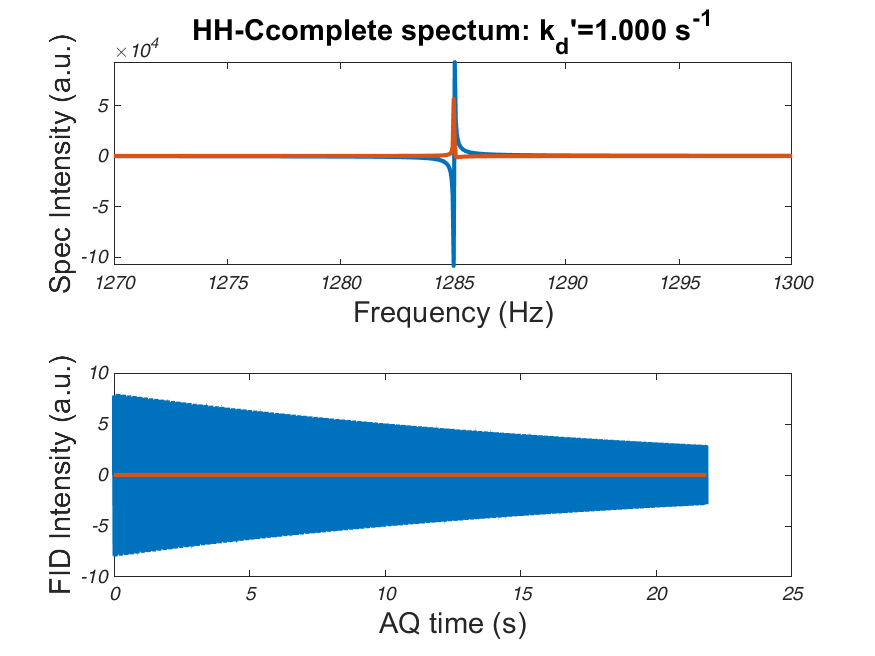

Supplement: Supplementary file 2 — jp3c01128_si_002.zip [file jp3c01128_si_002.zip › LIGHT_SABRE_2023_Paper_Pyruvate_JPCC/out/SLIC_SHEATH_SABRE_HH-C_1.png]

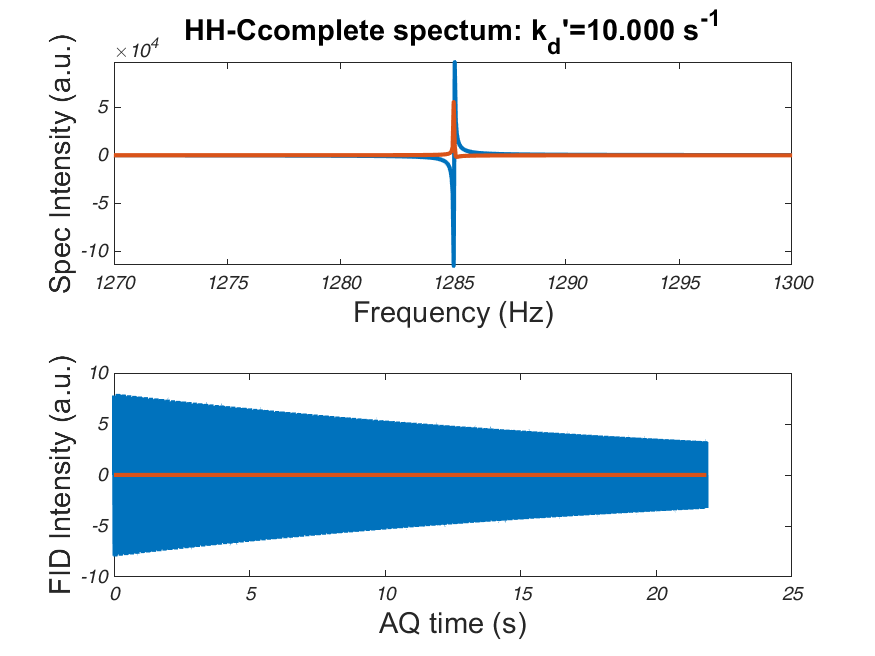

Supplement: Supplementary file 2 — jp3c01128_si_002.zip [file jp3c01128_si_002.zip › LIGHT_SABRE_2023_Paper_Pyruvate_JPCC/out/SLIC_SHEATH_SABRE_HH-C_10.png]

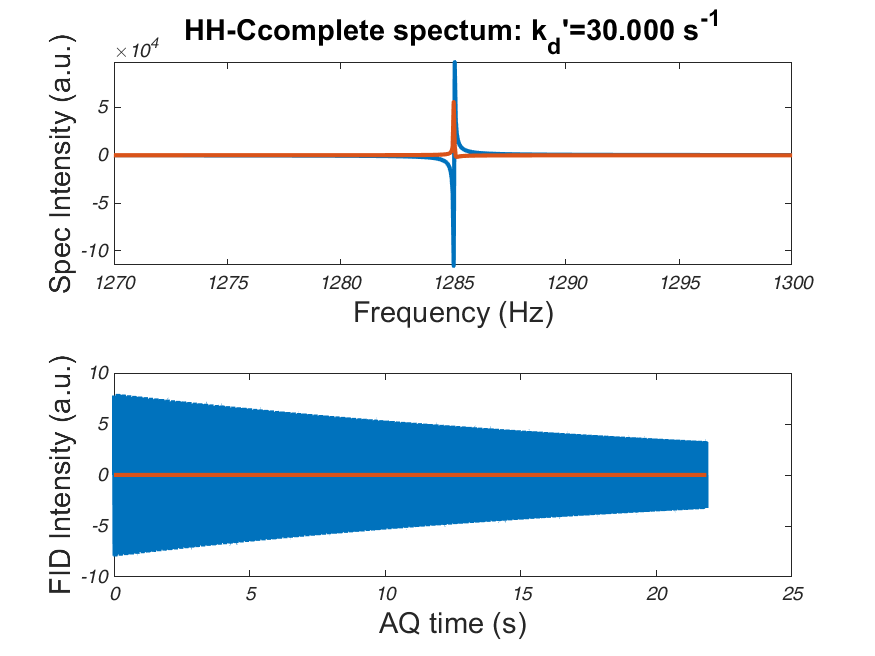

Supplement: Supplementary file 2 — jp3c01128_si_002.zip [file jp3c01128_si_002.zip › LIGHT_SABRE_2023_Paper_Pyruvate_JPCC/out/SLIC_SHEATH_SABRE_HH-C_30.png]
